# Wilms tumor primary cultures capture phenotypic heterogeneity and facilitate preclinical screening

**DOI:** 10.1016/j.tranon.2024.102263

**Published:** 2024-12-30

**Authors:** Lisa Götz, Jenny Wegert, Alireza Paikari, Silke Appenzeller, Sabrina Bausenwein, Christian Vokuhl, Taryn D. Treger, Jarno Drost, Christin Linderkamp, Dominik T. Schneider, Karen Ernestus, Steven W. Warman, Jörg Fuchs, Nils Welter, Norbert Graf, Sam Behjati, Rhoikos Furtwängler, Manfred Gessler

**Affiliations:** a Theodor-Boveri-Institute/Biocenter, Developmental Biochemistry, Julius-Maximilians-University Würzburg, Würzburg, Germany; b Comprehensive Cancer Center Mainfranken, University Hospital of Würzburg, Würzburg, Germany; c Section of Pediatric Pathology, Department of Pathology, University Hospital Bonn, Bonn, Germany; d Wellcome Sanger Institute, Hinxton, UK; e Department of Pediatrics, University of Cambridge, Cambridge CB2 0QQ, UK; f Cambridge University Hospitals NHS Foundation Trust, Cambridge CB2 0QQ, UK; g Princess Máxima Center for Pediatric Oncology, Utrecht, Netherlands; h Department of Pediatric Hematology and Oncology, Hannover Medical School (MHH), Hannover, Germany; i Clinic of Pediatrics, Klinikum Dortmund, University Witten/Herdecke, Germany; j Department of Pathology, University of Würzburg, Würzburg, Germany; k Clinic of Pediatric Surgery, Charité – University Hospital Berlin, Berlin, Germany; l Department of Pediatric Surgery and Pediatric Urology, University Children's Hospital, Tuebingen, Germany; m Department of Pediatric Hematology and Oncology, Saarland University Hospital, Homburg, Germany; n Pediatric Hematology and Oncology, Dep. of Pediatrics, Bern University Hospital, University of Bern, Inselspital, Switzerland

**Keywords:** Wilms tumor, Nephroblastoma, Organoid culture, Drug screening, MYCN

## Abstract

Wilms tumors (WT) are characterized by variable contributions of blastemal, epithelial and stromal elements, reflecting their diverse cellular origins and genetic drivers. In vitro models remain rare, despite a growing need to better characterize tumor biology and evaluate new treatments. Using three approaches, we have now established a large collection of long-term cultures that represent this diversity. Adherent WT cultures are predominated by stromal cells, 3D spheroids model blastema, and patient-derived organoid cultures of both tumor and healthy kidney tissue result in the preferential growth of epithelial cells. Adherent, spheroid and organoid cultures are also clearly distinguishable by their transcriptome. Preclinical drug screening experiments revealed sensitivity to a range of inhibitors, that are highly effective in other childhood solid tumors. Sensitivity was related to *MYCN* status, a marker associated with adverse outcome across human cancers including WT. The combination of the three culture techniques represents a promising tool to both explore tumor heterogeneity in vitro and to facilitate characterization of candidate driver genes, in order to improve treatment regimens in the future.

## Highlights

• 60 adherent, 30 organoid and 10 spheroid Wilms tumor cell cultures established.
• Culture conditions differentially support stromal, epithelial or blastemal cells.
• Different 2D/3D culture types represent distinct stages of kidney differentiation.
• Wilms tumor cultures are amenable to drug screening, organoids being most sensitive.
• *MYCN* gain or mutation as a promising target for treating high risk Wilms tumors.

## Introduction

Wilms tumor (WT), or nephroblastoma, is the most common renal tumor of early childhood. It is usually sporadic and unilateral, affecting about 1 in 10,000 children before the age of five [1]. WT is thought to arise from kidney precursor cells due to impaired or misguided development that can be caused by a variety of oncogenic drivers [2]. Inactivation of the Wilms tumor suppressor gene 1 (*WT1*), frequently in combination with activating *CTNNB1* mutations, was the first genetic cause to be unraveled [[3], [4], [5]]. Comprehensive screening efforts led to the discovery of an array of potential drivers, among them impairment of miRNA processing, *SIX1/2* and *TP53* mutations, amplification of *MYCN*, as well as epigenetic activation of *IGF2* [[6], [7], [8], [9], [10], [11], [12], [13], [14]].

WT is characterized by a high degree of histological inter- and intratumor heterogeneity. Untreated, it often presents with a triphasic histology consisting of blastemal, epithelial and stromal components, reminiscent of kidney development [15]. In general, the Société International d'Oncologie Pédiatrique (SIOP) guidelines recommend preoperative chemotherapy followed by nephrectomy and risk-adapted postoperative chemotherapy, supplemented by radiotherapy for higher stages [16]. Based on histological subtype after preoperative chemotherapy, the SIOP protocol stratifies tumors as low (necrotic), intermediate (stromal or epithelial predominant, triphasic, regressive, focal anaplastic) or high risk (blastemal predominant, diffuse anaplastic), to guide therapeutic regimens. Despite overall survival rates of > 90 %, long-term complications from chemo- and/or radiotherapy are not negligible and the outlook for relapsed or metastatic tumors is significantly worse, highlighting the need for better targeted or novel therapies.

Testing of novel agents has been limited as current preclinical models have severe shortcomings. Transgenic mouse models have only been reported for restricted genotypes, including *Wt1* ablation combined with *Igf2* dysregulation or *Lin28* overexpression [17,18]. Patient-derived xenograft models are difficult to handle, laborious and have only recently become available for limited testing [[19], [20], [21]]. While in vitro cell culture models appear attractive, they are often restricted to rare subtypes with *TP53* mutations [22]. Adherent primary cultures are predominated by stromal components, while epithelial or blastemal cells can be cultured only transiently or not at all [23,24]. We recently reported 3D floating spheroids, established from patient-derived material, that mimic the high-risk WT blastema and expand the repertoire of WT cultures [25]. However, long-term epithelial cultures could still not be obtained. To better model the histological diversity of WT, organoid culture conditions that have been shown to promote growth of healthy kidney tissue and pediatric renal tumors appear promising [26,27].

We have now established a large series of primary organoid cultures from multiple WT samples. We characterize their histology, genetics and gene expression patterns, comparing this with primary adherent and spheroid cultures. We further demonstrate their potential as preclinical models in drug screening experiments with both known and novel therapeutic agents.

## Materials/subjects and methods

### Patients and sample preparation

Tumor and normal kidney tissue was collected from the SIOP2001/GPOH and UMBRELLA SIOP-RTSG 2016 Wilms tumor studies (approved by the Ethikkommission der Ärztekammer des Saarlandes, reference numbers 136/01, 248/13 and 253/16). Clinical data were provided by the clinical study registry (N.G. and R.F.). All patients or their parents had provided informed consent for biobanking and biological studies. Tissue specimens were validated by histology of directly adjacent regions. Genomic DNA and RNA of tumor and kidney tissue was extracted using the Allprep DNA/RNA Mini kit (Qiagen, Hilden, Germany). Information on genomic driver mutations and nucleotide or copy number variants were generated in a larger independent study by whole genome sequencing (WGS) (Treger, Wegert et al., manuscript in preparation).

### Cell culture

Adherent and spheroid cultures were grown from patient-derived tissue within 24 h after surgery as described before [25]. For the establishment of organoid cultures, tissues were digested with collagenase I / DNase I (250 U/ml and 1 mg/ml in DMEM) and the supernatant, containing cells and small cell clusters, was pelleted and embedded in basement membrane extract (BME) type 2 (Cultrex PathClear®, bio-techne, Wiesbaden, Germany). Cultures were grown in DMEM high glucose/F-12 Ham (Merck, Darmstadt, Germany) containing GlutaMAX™ (Thermo Fisher Scientific, Darmstadt, Germany), 10 mM HEPES (pH 7.5), 50 U/ml penicillin, 50 µg/ml streptomycin, 1.5 % B-27 supplement (Thermo Fisher Scientific, Darmstadt, Germany), 50 ng/ml hEGF (Milteny Biotec, Bergisch Gladbach, Germany), 100 ng/ml hFGF10 [28], 10 µM Rho kinase inhibitor Y-27632 (Selleckchem, Köln, Germany), 1.25 mM N-acetylcysteine (Merck, Darmstadt, Germany), 125 ng/ml R-spondin [29], 5 µM A83–01 (Merck, Darmstadt, Germany) as described in Calandrini et al. [26]. Medium was changed every 2 - 3 days. For passaging (every 5 - 10 days), BME droplets containing organoids were dissolved with ice-cold medium, digested (37 °C, 5 - 10 min) with TrypLE™ Express (Thermo Fisher Scientific, Darmstadt, Germany) and cells were replated at a 1:2 split ratio. Dissociated cultures were cryopreserved in freezing medium (50 % DMEM high glucose/F-12 Ham, 40 % FCS, 10 % DMSO) and stored in liquid nitrogen.

### Genomic analysis

For mutation validation of primary cultures, corresponding genomic regions were amplified by PCR and sequenced by Sanger sequencing (Eurofins Genomics, Ebersberg, Germany). Copy number variations (CNV) were assessed by multiplex ligation-dependent probe amplification (MLPA) using the SALSA-MLPA-P380 kit (MRC Holland, Amsterdam, Netherlands). Loss of heterozygosity (LOH) was analyzed by PCR amplification of microsatellite loci in tumor and control samples (for primers see supplement table S4). The H19/IGF2 methylation status was determined using the MS-MLPA Probemix ME030 BWS/RSS (MRC Holland, Amsterdam, Netherlands).

### Immunohistochemistry

For histological analysis, organoids were extracted using Corning^Ⓡ^ Cell Recovery Solution (Corning, Corning, New York, USA), fixed in 4 % paraformaldehyde and embedded in paraffin. Hematoxylin and eosin (H&E) staining and immunohistochemistry of formalin-fixed, paraffin-embedded (FFPE) tumor tissue and organoids were performed as described before [25]. Sections were stained for Ki-67 (1:800, Agilent Technologies, Santa Clara, California, USA) and cytokeratin 7/8 (CAM5.2, 1:6, BD biosciences, Franklin Lakes, New Jersey, USA) with citrate buffer (pH 6.0) used for target retrieval.

### Transcriptome analysis

Total RNA sequencing of samples (RIN value > 7.50) was performed by BGI Tech (Hongkong) on the BGIseq500 NGS platform. Processing of raw sequencing data and calculation of PCA and differential expression were performed as previously described (Wegert et al. 2019) using a threshold of fpkm = 1. Further data analysis was done using the R2 Genomics Analysis and Visualization platform [30]. Two-tailed Student's t-test was used for statistical analysis. The BayesPrism algorithm [31] was used to estimate the cell type composition distribution in bulk RNA sequencing samples using single cell RNA sequencing data from fetal kidney and various WT subtypes [32]; Paikari et al., manuscript in preparation. Marker genes of the nephrogenic clusters are listed in the supplement (table S4).

### Drug screening

For organoid drug screening, single cell suspensions were seeded in 15 µl BME droplets in 48-well plates (10,000 cells/well) and organoids were grown for 3 days before treatment for 2 - 5 days depending on the compound and cell viability observed. Drug concentrations and treatment durations are shown in table S4. Spheroids were seeded at 13,000 (WK003-S), 8,000 (WK005-S and WK006-S) or 10,000 (WK040-S) cells/well in ultra-low attachment 96-well round bottom plates (Corning, Corning, New York, USA). Spheroid structures were grown for 4 days, followed by treatment for 2 days. Adherent cultures were grown to 80 % confluence in 96-well plates and treated for 2 days.

Cell viability was assessed by the CellTiter-Glo® Luminescent Cell Viability Assay kit (Promega, Madison, Wisconsin, USA). The absolute half maximal inhibitory concentration (IC_50_) was calculated based on nonlinear regression using GraphPad Prism version 9.0.1. Statistical analysis was done using the two-tailed Student's *t*-test or an analysis of variance in Origin Pro (version 2021b).

## Results

### Establishment of tumor and kidney organoid cultures

Organoid cultures of patient-derived tumor and normal kidney tissues were established from materials collected from 93 patients in the GPOH pediatric kidney tumor biobank (Table 1). Viable organoid cultures were generated from 39 primary and 5 relapse tumors. Highest success rates were seen with epithelial, blastemal and triphasic tumors. Failure (N = 49) was mainly due to a high percentage of regression or necrosis in the original tissue sample (table S1). Adjacent normal kidney organoids could be cultivated successfully in each of the 25 cases where normal tissue was available. With passaging every 5 - 10 days, organoids could be grown for several months without deterioration and cryopreserved with high recovery rates (> 70 % viable cells).

**Table 1:** Efficiency of tumor culture initiation.

	Spheroid	Adherent	Tumor organoid	Kidney organoid
Cultures started	99	98	93	25
No growth	88	19	49	0
Not informative*	1	8	4	0
Normal cells	0	11	10	25
Tumor derived	10	60	30	0

⁎ No tumor-specific alteration identified yet; validation was therefore not possible.

Tumor-specific genetic changes were used to validate organoid cultures as tumor-derived at passages 2 or 3. WTs have been characterized by either whole exome (WES) or whole genome sequencing (WGS), or by targeted sequencing of common driver genes in an independent study (Treger, Wegert et al., manuscript in preparation). The most common events underlying WT, namely loss of heterozygosity (LOH) at chromosome 11p15 and a loss of imprinting at the *IGF2* locus, were found using WES/WGS and methylation sensitive MLPA, respectively. Tumor-specific variants were used for validation, mainly by Sanger sequencing or MLPA (Table 2 and S1). A total of 30 out of 44 tumor organoid cultures faithfully represented tumor cells. Ten cultures were likely derived from interspersed normal kidney cells that may have been less affected by preoperative chemotherapy (table S1). In four instances, tumors lacked diagnostic alterations, precluding a final assignment. Organoids derived from corresponding healthy kidney tissue did not harbor these tumor-specific somatic alterations.

**Table 2:** Characteristics of organoid cultures.

Patient/ Culture	Sex	Age at diagnosis (months)	Tumor histology	Sample histology	Organoid morphology	Diagnostic genetic alterations
WK010	F	48	Regressive	Regressive	Compact	*MYCN* P44L, *CTNNB1* W383R
WK014	M	42	Triphasic	Regressive	Compact	*DROSHA* E993K + R1020*, 1p loss, trisomy 8, 11p loss
WK015	M	17	Stromal	Stromal	Mixed	*WT1* del exon6–9, *CTNNB1* S45Y, 11p LOH
WK029	F	40	Triphasic	Regressive	Compact	*DGCR8* E518K, *MYCN* gain, 1p loss, 1q gain, 14q loss
WK035	M	57	Triphasic	Blastemal	Compact	*BCOR* E1093*
WK036	M	8	Stromal	Stromal	Cystic	*WT1* Y295*, *CTNNB1* S45F, 11p LOH
WK036R			Blastemal	Triphasic	Compact	*WT1* Y295*, 1q gain, 11p LOH
WK040	F	61	Epithelial (DA)	Blastemal	Compact	*TRIM28* R487*, *MYCN* gain, *TP53* P68fs*76, multiple CNVs
WK042	M	44	Stromal (FA)	Necrosis	Compact	*AMER1* del, *MYCN* gain, 10p loss, 10q gain, 11p LOH
WK042R			Stromal	Stromal	Compact	*CTNNB1* S45Y, *MYCN* gain, 11p LOH

Tumor histology and histology of the starting material for cultures (sample histology) was validated by reference pathology (C.V.); DA, diffuse anaplasia; FA, focal anaplasia. Organoid cultures from relapses are indicated by an “R” suffix. CNV, copy number variation.

For in-depth characterization, tumor organoids from eight patients (WK010, WK014, WK015, WK029, WK035, WK036, WK040, WK042), two cultures derived from relapses (WK036R, WK042R) and five normal kidney cultures (WK012N, NK014, NK015, NK027, NK052) were chosen, as they represented the histological and genetic diversity of WT (Table 2, table S1).

**Fig. 1:**
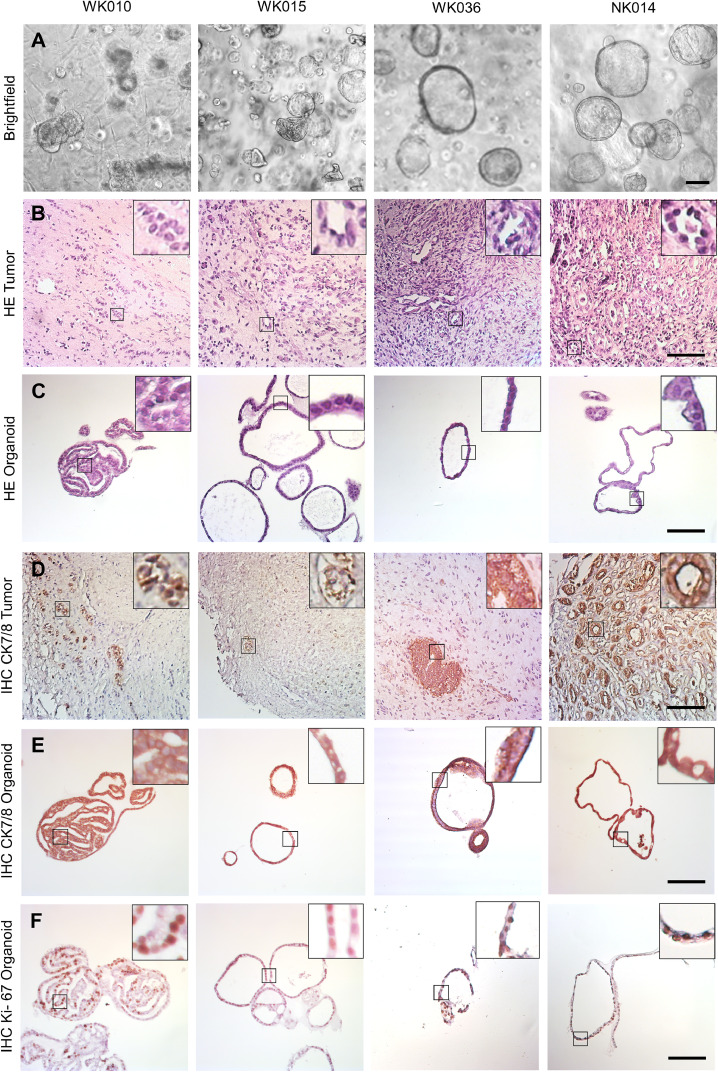
Morphology and proliferation of patient-derived WT and normal kidney organoids. Representative brightfield images of organoids cultured in BME (A). H&E staining of FFPE starting material (B) and organoids (C). Inserts show epithelial contributions. Cytokeratin 7/8 staining of tumor (D) and organoid (E) sections. Proliferation analysis with Ki-67 on organoid sections (F). Scale bar 100 µm.

There were clear morphological differences among the organoid cultures (Fig. 1, see figure S1 and S2 for additional examples). While kidney organoids invariably presented with cystic structures, tumor tissue predominantly yielded compact organoids (e.g. WK010) and rarely showed a cystic (e.g. WK036) or a mixed (e.g. WK015) morphology. In the latter, we observed a trend towards compact tumor organoids with long-term cultivation. Organoid morphology was not linked to the cellular composition of the original tissue used for culture initiation.

Cytokeratin 7/8 staining demonstrated a clear resemblance between epithelial tumor cells and organoid cultures (Fig. 1). Organoids were composed primarily of epithelial cells, except for WK014 that exhibited a minor contribution of blastemal-like cells (5 %). Sporadic outgrowths of stromal elements that exhibited fibroblast-like morphology were depleted during cultivation (see panel A of WK010 in Fig. 1). Proliferation rate, as assessed by Ki-67 staining, was high in compact (50 % positive cells) and cystic (70 - 80 %) tumor organoids, as well as in normal kidney cultures (70 - 80 %).

### Establishment of adherent and spheroid cultures

In parallel to organoid cultures, material from the same minced tumor biopsy was used to initiate spheroid and adherent cultures. Stromal adherent tumor cultures were obtained in most cases, provided that the starting material contained viable cells (60/98). The proportion of truly tumor-derived cultures was also higher (60/71) (Table 1). In contrast, long-term proliferating spheroid cultures that resemble WT blastema were rarely obtained (10/99). Among the adherent cultures there were five cultures that later turned out to be derived from non-Wilms renal tumors according to reference pathology (2 CMN, 2 CCSK, 1 CN with the typical *EGFR*-ITD, *BCOR*-ITD and *DICER1* mutations, table S1). None of these tumors yielded viable organoid or spheroid cultures indicating that the conditions may have to be optimized further for non-Wilms tumors. Together with prior efforts we have generated a collection of 25 normal kidney organoids, 30 tumor organoids, 10 tumor spheroids and > 90 adherent cultures from WT, each validated by genetic marker analysis [24,25].

These culture types were only partially convertible into each other. For spheroids, we have shown previously that they are able to grow adherently and can be transformed back into spheroids even after several passages [25]. Attempts to propagate established organoid cultures as free-floating spheres and vice versa failed, however. Similarly, organoid cells could not be propagated as adherent cultures without matrix embedding.

### Transcriptome analysis of primary WT cultures

To better classify primary WT culture types, we compared the transcriptome profiles of 15 organoid (8 primary tumor, 2 relapse, 5 normal kidney), 8 spheroid (including 2 later passages) and 10 adherent cultures that were derived from a total of 19 patients. All samples were harvested at passage 2 - 4 unless otherwise indicated. For one primary (WK010) and two pairs of primary and relapse tumors (WK036(R), WK042(R)), both organoid and adherent cultures were available.

**Fig. 2:**
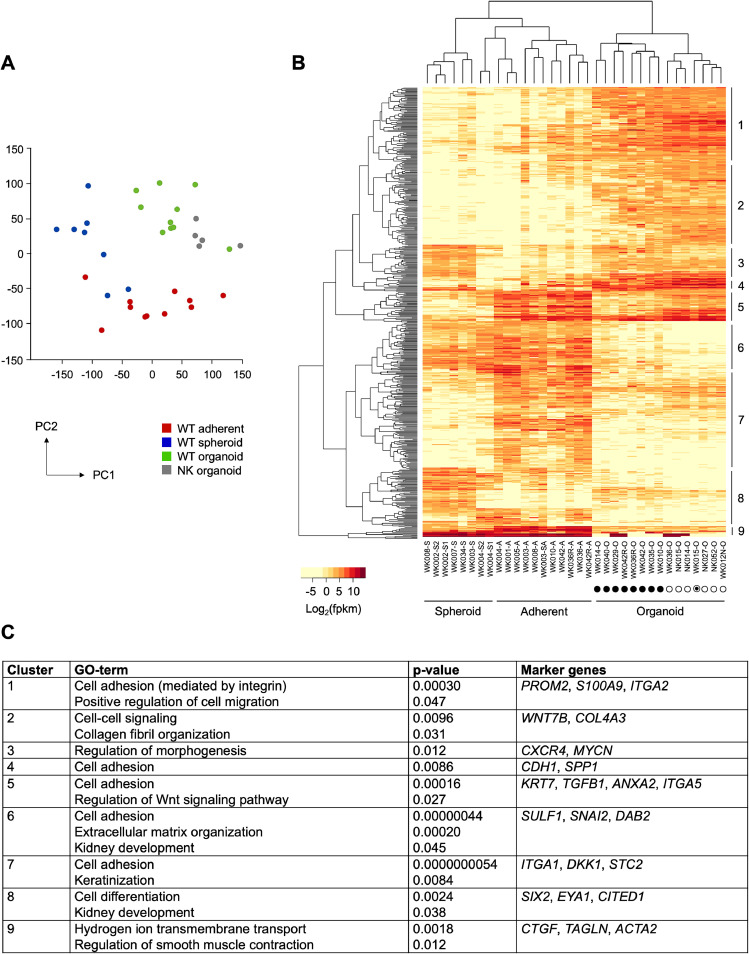
Expression profile of Wilms tumor cultures and normal kidney controls. Separation of adherent, spheroid and organoid WT cultures and normal kidney organoids based on the principal component analysis (A). Unsupervised clustering of organoid, spheroid and adherent cultures from tumors (WK) and kidney (NK) based on the 500 most differentially expressed genes with the highest PC loadings (B). Clusters 1 - 9 are annotated on the right. Suffixes denote culture type (*S* = spheroid, *A* = adherent, *O* = organoid, S1/S2 = early/late passage, SA = adherent derived from spheroid). Symbols encode organoid morphology (● = compact, *O* = cystic, ⦿ = mixed). Data for samples WK001, WK002, WK003, WK004, WK005, WK007, WK008 are from Wegert et al. (2020). The most relevant GO-terms found for clusters of differentially expressed genes are listed (C). Highly overlapping GO-terms were combined for brevity (for a comprehensive analysis see supplement table S2).

Principle component analysis (PCA) separated the three culture types (Fig. 2A) which was supported by unsupervised clustering based on the 500 most differentially expressed genes (Fig. 2B, table S2). The organoids were clearly distinct, while spheroid and adherent cultures showed more similarities. Pairs of cultures derived from the same tumor were grouped according to culture type and not their initial source (e.g. adherent vs. organoid cultures of WK036/WK036R, WK042/WK042R, or WK010). Normal kidney organoids showed strong similarities with tumor organoids. The organoid culture WK012 was derived from completely necrotic tumor material, it lacked driver mutations (table S1) and clustered together with other kidney cultures, supporting its classification as normal cells and subsequent labeling as WK012N.

Clusters of differentially expressed genes were functionally annotated using DAVID and WT culture types could be clearly distinguished based on the expression levels of epithelial, stromal and blastemal/kidney progenitor markers [33] (david.ncifcrf.gov; Fig. 2C and table S2). Clusters 1 and 2 were unique to tumor and kidney organoid samples, characterized by genes of extracellular matrix organization, integrin and cell-cell signaling. In line with their epithelial histology, organoids expressed a set of epithelial marker genes (clusters 1, 4, 5), including *CDH1, KRT7* and *SPP1*, which were not found in spheroids and minimally represented in adherent cultures. Stromal marker genes (e.g. *SULF1, ITGA1, DKK1*) were mainly restricted to the adherent cultures (clusters 6, 7), whilst spheroids showed a high expression of self-renewing nephron progenitor markers, including *SIX2, EYA1* and *CITED1* (cluster 8).

### Organoids differ according to morphology and *MYCN* status

**Fig. 3:**
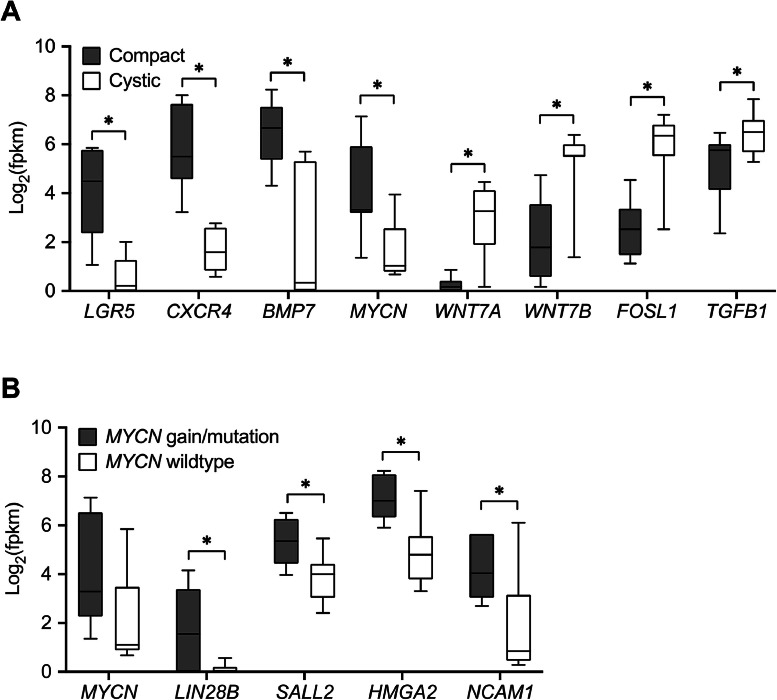
Subgroups of organoids defined by morphology and *MYCN* pattern. Examples of genes upregulated in either compact or cystic cultures (A). High *MYCN* expression levels in tumor organoids harboring *MYCN* alterations were accompanied by a set of coregulated genes (B).

Unsupervised clustering further separated organoid cultures according to their growth pattern. Cystic and mixed tumor organoids were intermingled with normal kidney organoids, while compact ones were clearly separated (Fig. 2B). Genes of the BMP signaling pathway, extracellular matrix organization and homophilic cell adhesion (e.g. *LGR5, CXCR4, BMP7, MYCN*) were highly expressed in compact organoids (Fig. 3A). In cystic cultures, cell differentiation genes were predominant (e.g. *WNT7A, WNT7B, FOSL1, TGFB1*), while the mixed culture (WK015) exhibited intermediate expression levels.

The differential expression of *TGFB1* led us to investigate the role of medium supplementation with the TGF-β receptor inhibitor A83–01. Indeed, addition or removal of A83–01 led to a switch in morphology of the mixed culture WK015 towards either compact or cystic structures, respectively (figure S3).

*MYCN*, a key driver of several pediatric tumors and a prominent negative prognostic marker in Wilms tumors [14], showed differential expression, with elevated levels in compact organoids. In our cohort, WK029, WK040, WK042 and WK042R had a *MYCN* copy number gain (copy number of 3), while WK010 had an oncogenic P44L mutation. These *MYCN* altered organoids were characterized by a whole set of co-regulated genes (Fig. 3B) and many of these are typically found in immature nephrogenic lineages [18,34]. The corresponding adherent cultures from the same patients (WK010, WK042, WK042R) showed much lower and thus less informative *MYCN* expression as is typical for WT stroma (figure S4). This difference is apparently not compensated by expression of the paralog *MYC*.

### WT cultures represent different kidney lineages

**Fig. 4:**
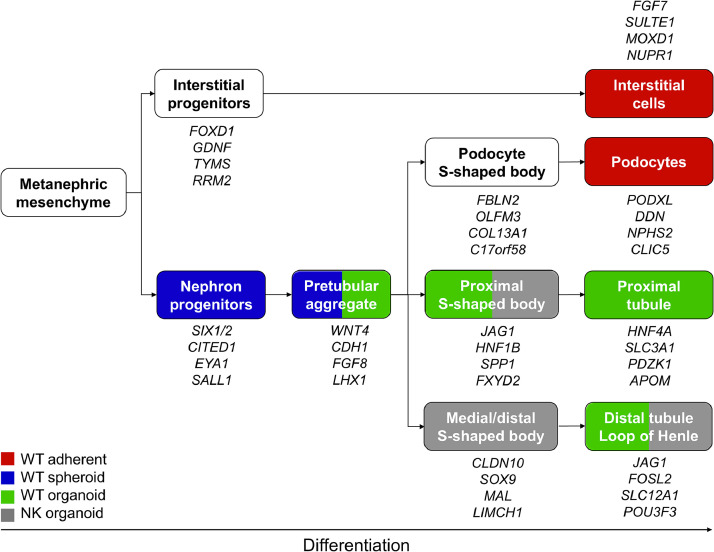
Primary WT cultures correspond to different kidney lineages. Adherent, spheroid and organoid cultures differentially resemble interstitial, early mesenchymal or epithelial renal cell types. Main marker genes for classifications are listed. For a complete list of markers see table S4. Cut-off for color coding: ≥ 3 Samples > 10 % proportion (in individual patients).

Deconvolution of bulk transcriptome data based on single cell RNA sequencing from fetal kidney and WT (A.P. et al., manuscript in preparation) revealed diverse nephrogenic populations in organoid, spheroid and adherent WT cultures. While adherent cultures were most similar to interstitial cells, spheroids mainly corresponded to nephron progenitors and to early pretubular aggregates. Organoids reflected various stages of advanced nephrogenesis, from pretubular aggregates and S-shaped bodies up to epithelia of proximal or distal tubules and the loop of Henle (Fig. 4). Differences between compact and cystic organoids were again apparent: while compact organoids mainly mapped to early pretubular aggregates, cystic tumor and normal kidney cultures expressed signatures of more mature S-shaped bodies and their derivatives at substantial higher levels (table S2).

### Validation of primary WT cultures for drug screening

**Fig. 5:**
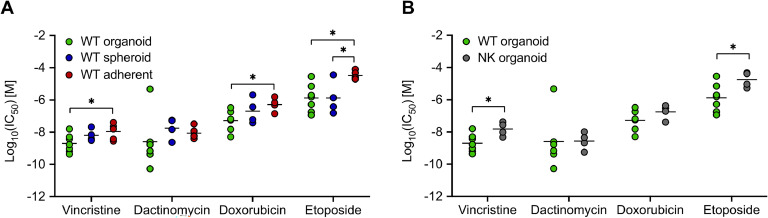
Validation of primary WT cultures for use in drug screening. Organoid (WK010, WK035, WK036, WK036R, WK040, WK042, WK042R), spheroid (WK002, WK003, WK004, WK034) and adherent (WK010, WK036, WK036R, WK042, WK042R) tumor cultures were treated with vincristine, dactinomycin, doxorubicin and etoposide (Etoposide) and IC_50_ values are shown by culture type (A). Tumor organoids resulted in lower IC_50_ values than kidney-derived cultures (NK014, NK015, NK027, NK052) (B). For individual IC_50_ values see table S3. *p < 0.05.

To evaluate the suitability of primary WT cultures for preclinical drug screening, we tested their response to current chemotherapeutic regimens. These include dactinomycin and vincristine, with the addition of doxorubicin or etoposide in high-risk cases. For organoid, spheroid and adherent cultures, the treatment at clinically relevant concentrations [35] resulted in strong effects on cell viability (Fig. 5, for IC_50_ values see table S3). Compared to adherent cells, organoids were significantly more sensitive to all four agents, while spheroid cultures showed an intermediate sensitivity. Differences between culture types may be due to intrinsic variation in apoptosis resistance depending on cellular contacts, cell polarity, and for spheroids also accessibility. There were marked patient-specific differences in the IC_50_ values, particularly for spheroids and organoids. Among organoids, more differentiated cystic kidney organoids showed a trend toward lower sensitivity (Fig. 5B).

### Inhibitors that target tumor cells in a MYCN-dependent manner

Successful therapy of high-risk WT requires novel approaches beyond conventional cytotoxic regimens. Targeting activated MYCN has become a focus across childhood and adult cancer [36]. The negative prognostic value of oncogenic *MYCN* in WT [14] makes it an attractive target for inhibitors that have shown promising results in other pediatric malignancies.

**Fig. 6:**
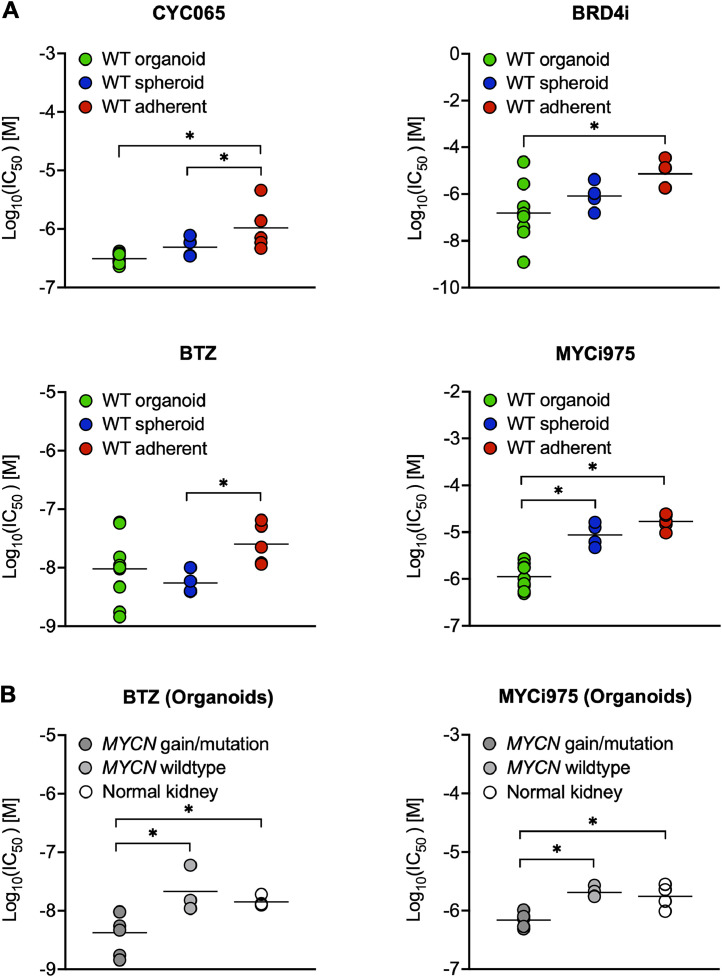
Inhibitors known to target tumor cells in a *MYCN*-dependent manner showed efficacy in WT cultures. WT cultures (organoid: *n* = 10, spheroid: *n* = 4, adherent: *n* = 5) were treated with CYC065, BRD4i, bortezomib (BTZ) and MYCi975 (A). Sensitivity as measured by absolute log(IC_50_) values was lower in adherent cultures. Response of organoids to bortezomib (BTZ) and MYCi975 (B) was significantly stronger if they harbored a *MYCN* alteration (genomic gain or activating mutation, *n* = 6) compared with wildtype tumor (*n* = 4) and normal kidney (*n* = 4) cultures. For individual IC_50_ values see table S3. *p < 0.05.

The CDK9/2 inhibitor CYC065 that selectively targets *MYCN*-amplified neuroblastoma in vitro [37], caused growth arrest after 48 h and cell death in WT cultures upon prolonged treatment for 5 days. This effect was especially seen in WT organoids and was less pronounced in spheroid and adherent cultures (Fig. 6A). Treatment with a BRD4 inhibitor (BRD4i), reported to reduce *MYCN* levels in WT cell lines [38], was also effective in our WT cultures, again most strongly in spheroids and organoids (Fig. 6A). The effects of both drugs were independent of *MYCN* gain or mutation, however.

The FDA-approved proteasome inhibitor bortezomib (BTZ) efficiently targets neuroblastoma cell lines in a *MYCN*-dependent manner in vitro [39]. In our cultures, treatment with BTZ strongly reduced cell viability. Spheroids that highly expressed *MYCN* and organoid cultures were more affected than adherent cultures (Fig. 6A). The variation of IC_50_ values in organoids was linked to *MYCN* status. Cultures with *MYCN* gain (WK029, WK040, WK042, WK042R) or an activating mutation (WK010), showed a significantly stronger sensitivity to BTZ than tumor organoids with wildtype *MYCN*. The combination of low-dose BTZ and the HDAC inhibitor vorinostat (SAHA) that synergistically induces apoptosis in vitro [40], showed a significant additive effect in organoids with high *MYCN* expression, particularly in the relapsed case (WK042(R), figure S5).

The small molecule MYC inhibitor (MYCi975) that disrupts MYC/MAX dimers and induces MYC degradation in diverse tumor cell lines [41] likewise reduced cell viability of WT cultures, with organoids being most sensitive (Fig. 6B). *MYCN* gain and the activating P44L mutation were again associated with a significantly stronger response to MYCi975 compared to other tumor organoids (Fig. 6B).

## Discussion

Functional analysis of oncogenic drivers and the evaluation of new treatment strategies in WT require preclinical models that represent the genetic and morphological heterogeneity. Here, we show that the combination of adherent, spheroid and organoid primary cultures is a promising tool for preclinical drug screening, as it covers stromal [24], blastemal [25] and epithelial WT elements. Previously described adherent and spheroid primary cultures were not able to model epithelial WT. All 2D epithelial cultures were short-lived and underwent senescence [24]. Therefore, an additional culture protocol was used to establish patient-derived WT and kidney organoids based on a protocol originally published by Calandrini et al. [26].

The overall efficacy of culture initiation was compromised in our case by regressive or necrotic tissue in many tumor samples from preoperative chemotherapy administered as per treatment guidelines. Logistic factors in the Germany-wide transfer of tumor material may also have decreased cell viability and thus reduced the efficacy of culture establishment. Enrichment of viable cells from clinical specimens may address this issue in the future. Nevertheless, the resulting organoid collection reflects known WT driver mutations including alterations in *WT1, CTNNB1, DGCR8, BCOR*, and *TRIM28*, as well as *MYCN* copy number gains [6,8,12,13,42]. This diversity suggests that tumor organoids provide an unbiased representation of the genetic heterogeneity of WT.

Both tumor and kidney organoid cultures were histologically classified as predominantly epithelial, consistent with data on organoids derived from various epithelial tissues, which may result from supportive medium composition and attachment options [43]. In some cases, only a small percentage of epithelial cells was present, which nevertheless gave rise to epithelial WT organoids, regardless of the histology of the original tumor sample. Therefore, our tumor cultures will certainly not represent the cellular composition of the original tumor, but rather reflect the result of a selection during earlier passages.

Although stromal elements were present in early organoid passages, their presence was variable, and they were gradually lost. The long-term coexistence of all three WT cell types as reported in two of the cultures by Calandrini et al. [26] was not observed in our cultures. It may strongly depend on culture conditions to avoid either gradual overgrowth of any one lineage, or loss of e.g. stromal cells. Differences in handling, either in passaging or during staining may allow for preferential loss of unattached stromal cells. On the other hand, discrepancies may also result from cell type classification. In particular, the proposed blastemal markers *NCAM* and *SIX2* are still present in epithelial progenitors, making a clear demarcation difficult [44]. Based on our comparative analyses with spheroids representing true blastema, we categorized organoids with a persistent but lower *NCAM* expression as early epithelial representatives rather than blastemal-like cells in our study.

Tumor organoid cultures either displayed a compact or cystic morphology with a preference for compact structures during long-term cultivation, while mixed cultures were rare. Compact tumor organoids exhibited a higher expression of genes involved in WNT-mediated maintenance of nephron progenitors (e.g. *LGR5, MYCN, HMGA2, LIN28B, NCAM, WNT4, BMP7*). On the other hand, markers of the advanced nephrogenesis (e.g. *WNT7A, WNT7B, TGFB1*) were found predominantly in cystic cultures, suggesting that the two morphological groups represent different developmental stages [45,46]. Selection forces during extended growth in vitro and inhibition of TGF-β receptor signaling may in part explain this morphological switch.

Adherent, spheroid and organoid cultures not only differentially favored growth of the stromal, blastemal or epithelial WT components, but they could be distinguished based on their expression profile. Adherent cultures highly expressed cell adhesion genes, as well as those of heterologous differentiation (e.g. muscle), typical for WT stroma. Genes of the early kidney development were mainly found in spheroids resembling nephron progenitor cells. Organoids exhibited an epithelial expression pattern representative of more differentiated nephrogenic lineages (e.g. pretubular aggregates, S-shaped bodies, proximal and distal tubules).

In most cases, only one or two of the conditions were successful in establishing cultures from a given tumor. This suggests that factors such as original cell type composition and heterogeneity, the differential viability or the genetic status of the original biopsy material may favor one type of culture over the other. Furthermore, the number of starting cells required appears to be quite different, with spheroids needing a high number of blastemal cells for successful initiation. The culture conditions likely also exert a selection pressure on cell types, resulting in these separate, and internally rather uniform states in terms of morphology and gene expression, which only in a few instances can be converted into one another. Parallel approaches will therefore maximize the success rates of future cultivation efforts.

There is a strong need for improved therapeutic regimens in high-risk WT. Targeted therapy is a promising alternative to classical chemotherapy to overcome resistance and to mitigate severe side effects [47]. Since *MYCN* dysregulation is associated with anaplasia and a worse overall prognosis, targeting MYCN is a promising option [14,48,49]. Our results strongly suggest that further testing of proteasome inhibitors, in particular bortezomib, is warranted. It is already approved for second-line treatment of multiple myeloma and mantle-cell lymphoma [50]. Bortezomib preferentially affected organoid cultures with *MYCN* gain or the activating P44L mutation. The lethal response in our cultures occurred in the nanomolar concentration range, well below the recommended doses in patients and comparable to *MYCN*-driven neuroblastoma cell lines [39]. Especially in the case of *MYCN*-amplified relapsed WT, the combination of bortezomib with an HDAC inhibitor could improve overall survival rates through a synergistic effect that has already been described in other tumor entities [40]. The effect of direct MYCN inhibition by an experimental small molecule inhibitor provides further support that targeting MYCN could play an important role in the treatment of high-risk WT.

Although time constraints are likely to limit the use of patient-specific cell cultures for personalized drug testing, our primary WT cultures represent a valuable tool for efficient prospective screening of available and approved or novel drugs. Our current results already suggest new treatment rationales such as MYCN targeting and the use of proteasome inhibitors and its combination with HDACi in these highly proliferative tumors.

## CRediT authorship contribution statement

**Lisa Götz:** Writing – review & editing, Writing – original draft, Visualization, Validation, Methodology, Investigation, Formal analysis, Data curation. **Jenny Wegert:** Writing – review & editing, Validation, Supervision, Resources, Methodology, Investigation, Formal analysis, Data curation, Conceptualization. **Alireza Paikari:** Writing – review & editing, Software, Investigation, Formal analysis, Data curation. **Silke Appenzeller:** Writing – review & editing, Software, Methodology, Formal analysis, Data curation. **Sabrina Bausenwein:** Writing – review & editing, Validation, Resources, Investigation. **Christian Vokuhl:** Writing – review & editing, Validation, Methodology, Investigation, Formal analysis, Data curation. **Taryn D. Treger:** Writing – review & editing, Software, Methodology, Investigation, Formal analysis, Data curation. **Jarno Drost:** Writing – review & editing, Resources, Methodology. **Christin Linderkamp:** Writing – review & editing, Resources. **Dominik T. Schneider:** Writing – review & editing, Resources. **Karen Ernestus:** Writing – review & editing, Validation, Resources, Methodology. **Steven W. Warman:** Writing – review & editing, Resources. **Jörg Fuchs:** Writing – review & editing, Resources. **Nils Welter:** Writing – review & editing, Resources, Data curation. **Norbert Graf:** Writing – review & editing, Resources, Funding acquisition, Formal analysis, Data curation. **Sam Behjati:** Writing – review & editing, Software, Resources, Funding acquisition. **Rhoikos Furtwängler:** Writing – review & editing, Resources, Formal analysis, Conceptualization. **Manfred Gessler:** Writing – review & editing, Writing – original draft, Visualization, Validation, Supervision, Resources, Project administration, Methodology, Investigation, Funding acquisition, Formal analysis, Data curation, Conceptualization.

## Declaration of competing interest

The authors declare that they have no known competing financial interests or personal relationships that could have appeared to influence the work reported in this paper.

## Data Availability

All data needed to evaluate the conclusions in the paper are present in the paper and the Supplementary Materials.

## Appendix Supplementary materials

Figure S1: Histological analysis of tumor organoids (not characterized in Figure 1). H&E staining of FFPE specimens of starting material and organoids on the left. IHC for the proliferation marker Ki-67 is shown on organoid sections. Representative brightfield images of tumor organoids cultured in BME on the right. Scale bar 100 µm. Image, image 1

Figure S2: Histological analysis of kidney organoids (not characterized in Figure 1). H&E staining of FFPE specimens of starting material and organoids on the left. IHC for the proliferation marker Ki-67 is shown on organoid sections. Representative brightfield images of kidney organoids cultured in Matrigel on the right. Scale bar 100 µm. Image, image 2

Figure S3: Morphology of organoids can be controlled by TGF-ß inhibition with A83–01. Selection for compact structures in the morphological mixed culture WK015 was prevented by growing organoids in the absence of the TGF-β receptor inhibitor A83–01 and morphology was shifted towards cystic structures. This effect could be reversed by supplementing the organoid growth medium again with A83–01. Scale bar 100 µm. Image, image 3

Figure S4: Expression of MYC family members in primary WT cultures. *MYCN* expression was significantly higher in organoids and spheroids and lower in adherent WT cultures, while *MYC* showed no culture type-dependent differences in expression. **p* < 0.05. Image, image 4

Figure S5: Proteasome inhibition combined with HDAC inhibitor treatment. Treating *MYCN* amplified (WK042, WK042R), *MYCN* wildtype (WK036) and normal kidney (NK015) organoid cultures with bortezomib (Bortezomib) and SAHA revealed an additive effect in the cultures derived from a relapsed WT patient (WK042, WK042R). **p* < 0.05. Image, image 5

Supplementary table S1: Overview of WT cultures. Clinical and molecular data of tumor and kidney cultures and their growth pattern. Image, application 6

Supplementary table S2: RNA expression data for all cell cultures. (1) Top 500 most differentially expressed genes in WT cultures, (2) GO term annotation of 500 most differentially expressed genes, (3) representation of nephrogenic clusters in WT cultures. Image, application 7

Supplementary table S3: IC_50_ values of drug screenings in WT cultures. Image, application 8

Supplementary table S4: Supplementary methods. Listing of PCR primers, markers used in RNA deconvolution and drugs used for screening. Image, application 9
