# Supplementary figures and images for: Wilms tumor primary cultures capture phenotypic heterogeneity and facilitate preclinical screening
Source: Transl Oncol. 2024 Dec 30;52:102263. doi: 10.1016/j.tranon.2024.102263 (PMC11750297; doi:10.1016/j.tranon.2024.102263)

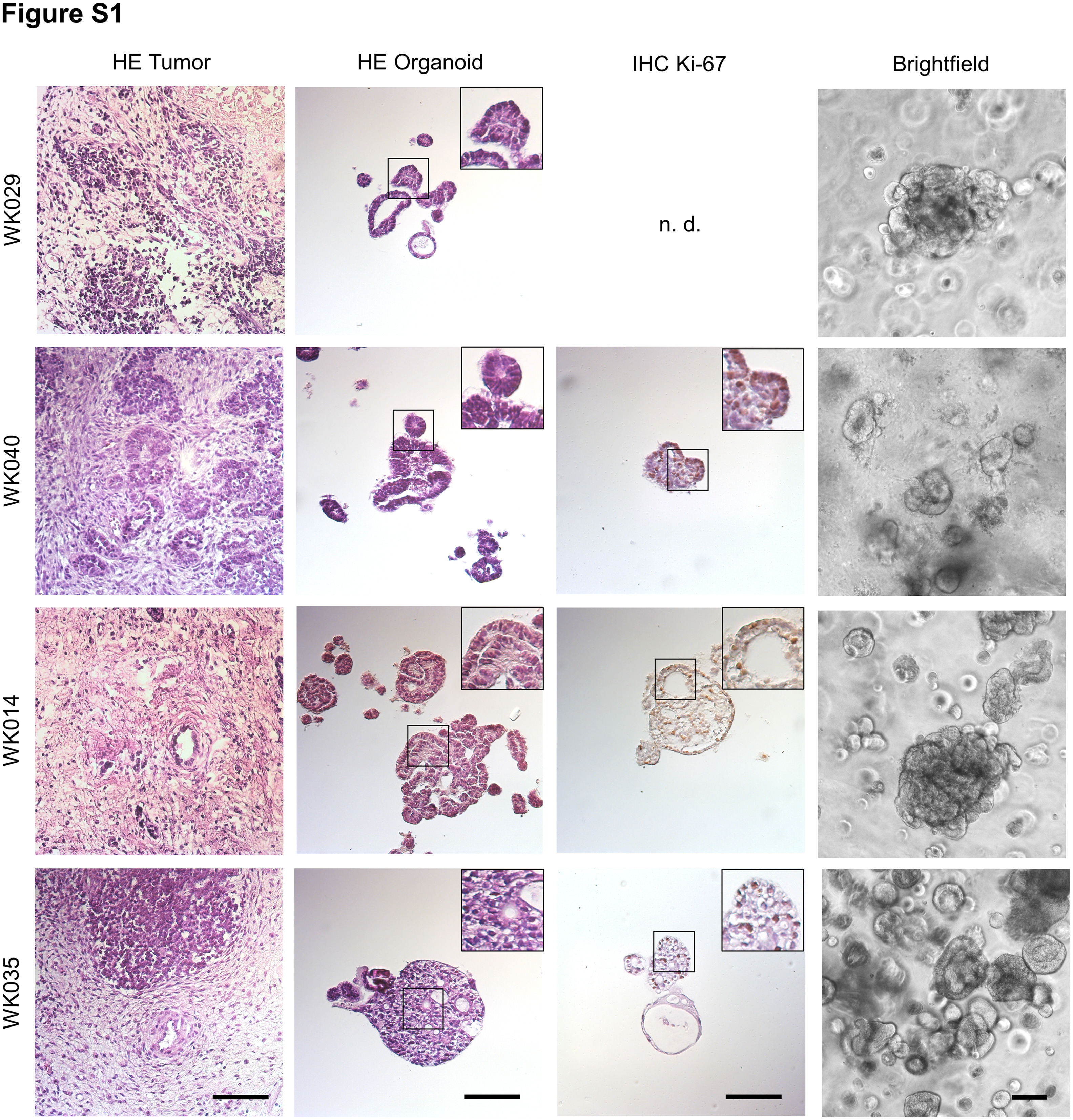

Supplement: Supplementary file 1 — Figure S1: Histological analysis of tumor organoids (not characterized in Figure 1). H&E staining of FFPE specimens of starting material and organoids on the left. IHC for the proliferation marker Ki-67 is shown on organoid sections. Representative brightfield images of tumor organoids cultured in BME on the right. Scale bar 100 µm. [file mmc1.jpg]

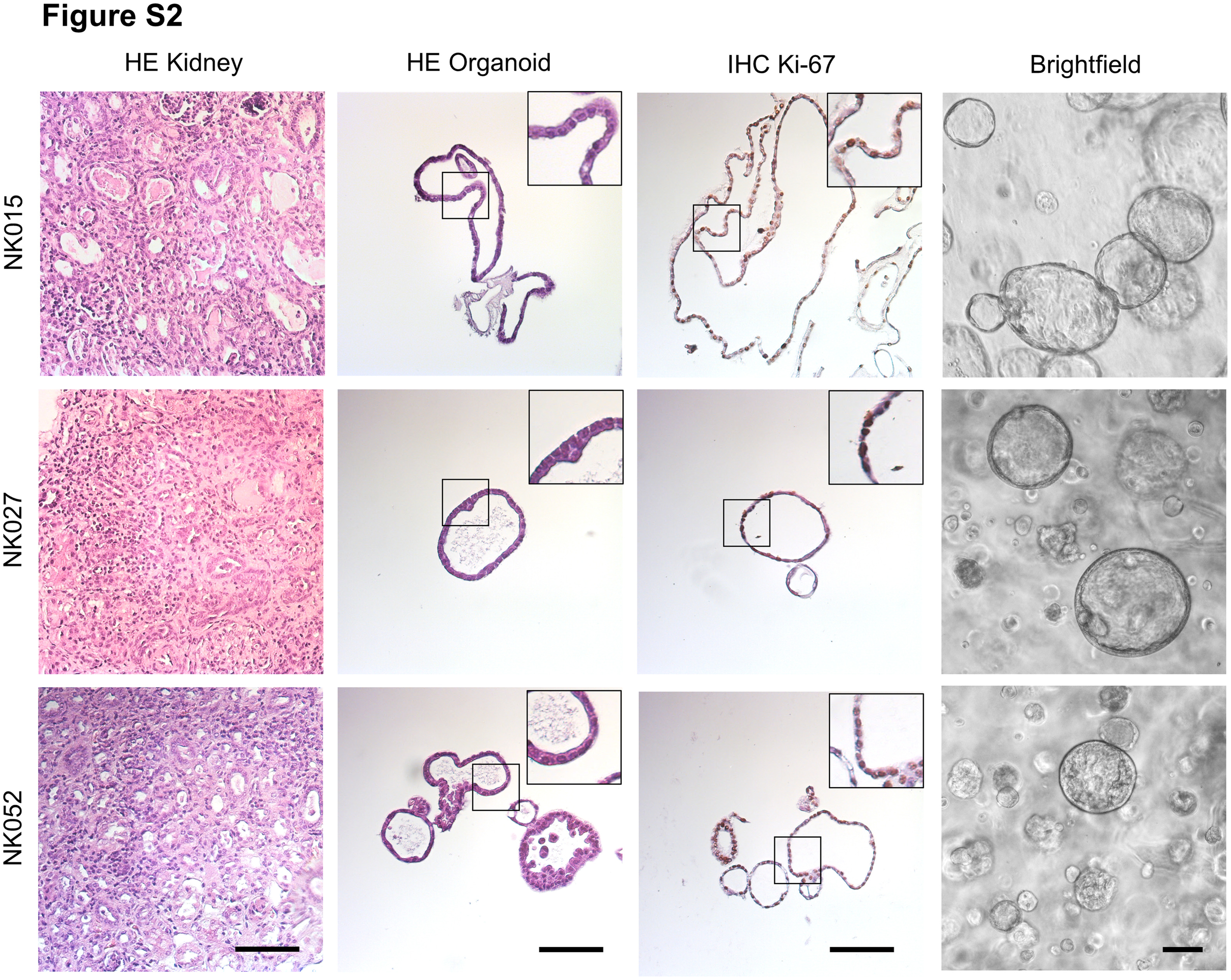

Supplement: Supplementary file 2 — Figure S2: Histological analysis of kidney organoids (not characterized in Figure 1). H&E staining of FFPE specimens of starting material and organoids on the left. IHC for the proliferation marker Ki-67 is shown on organoid sections. Representative brightfield images of kidney organoids cultured in Matrigel on the right. Scale bar 100 µm. [file mmc2.jpg]

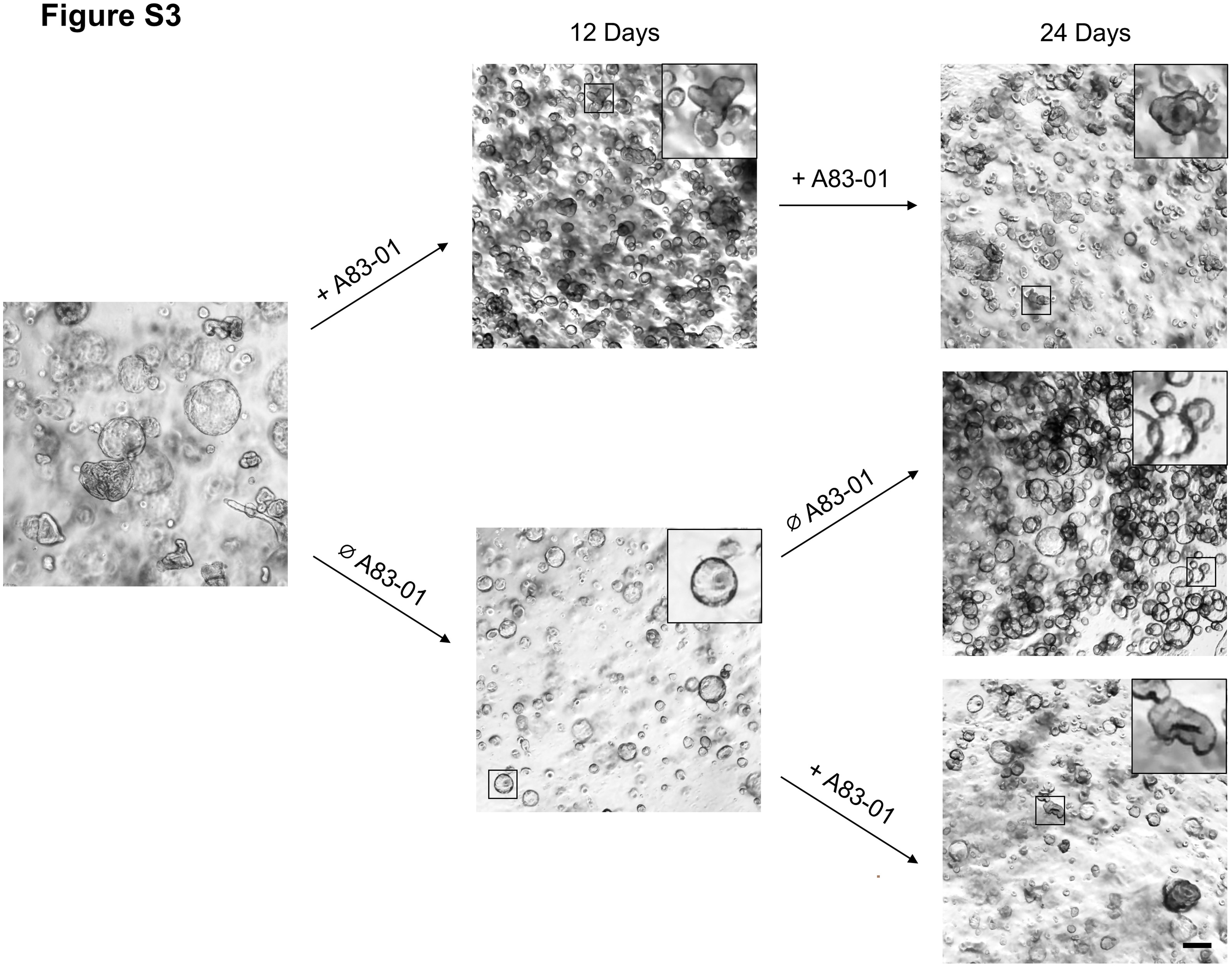

Supplement: Supplementary file 3 — Figure S3: Morphology of organoids can be controlled by TGF-ß inhibition with A83–01. Selection for compact structures in the morphological mixed culture WK015 was prevented by growing organoids in the absence of the TGF-β receptor inhibitor A83–01 and morphology was shifted towards cystic structures. This effect could be reversed by supplementing the organoid growth medium again with A83–01. Scale bar 100 µm. [file mmc3.jpg]

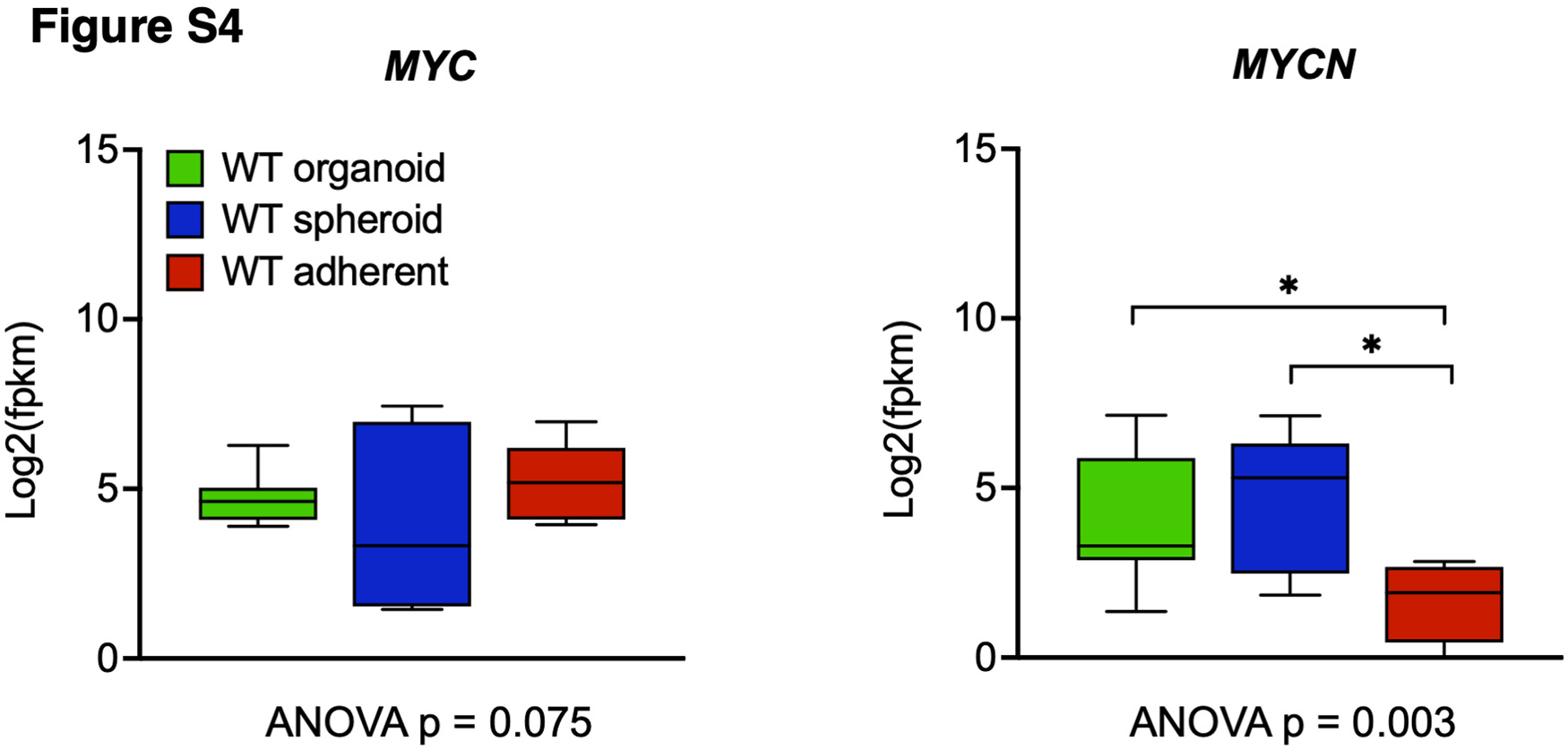

Supplement: Supplementary file 4 — Figure S4: Expression of MYC family members in primary WT cultures. MYCN expression was significantly higher in organoids and spheroids and lower in adherent WT cultures, while MYC showed no culture type-dependent differences in expression. *p < 0.05. [file mmc4.jpg]

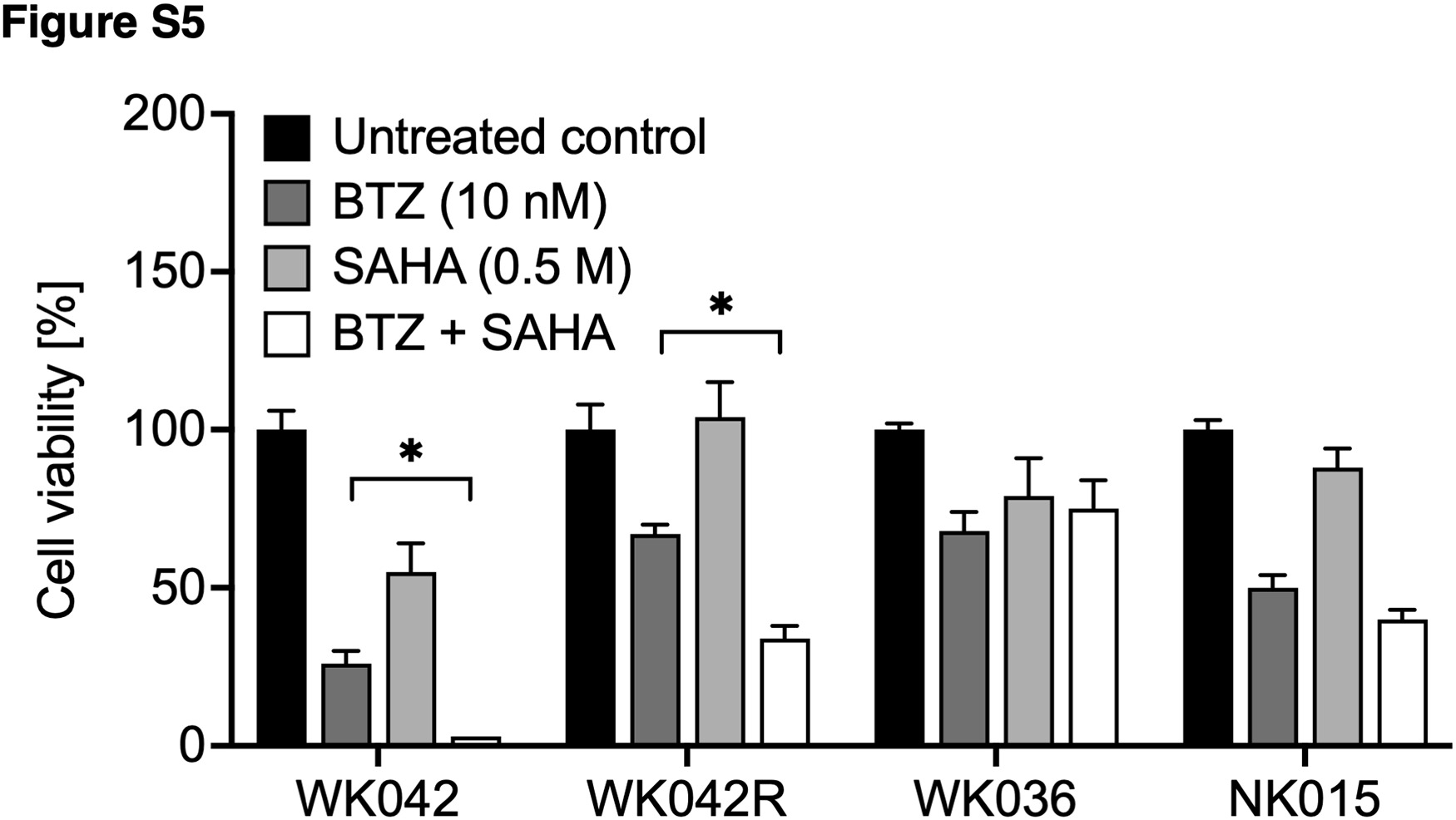

Supplement: Supplementary file 5 — Figure S5: Proteasome inhibition combined with HDAC inhibitor treatment. Treating MYCN amplified (WK042, WK042R), MYCN wildtype (WK036) and normal kidney (NK015) organoid cultures with bortezomib (Bortezomib) and SAHA revealed an additive effect in the cultures derived from a relapsed WT patient (WK042, WK042R). *p < 0.05. [file mmc5.jpg]
